# Evolution of Diagnostic and Forensic Microbiology in the Era of Artificial Intelligence

**DOI:** 10.7759/cureus.45738

**Published:** 2023-09-21

**Authors:** Anwita Mishra, Salman Khan, Arghya Das, Bharat C Das

**Affiliations:** 1 Department of Microbiology, Mahamana Pandit Madan Mohan Malviya Cancer Centre and Homi Bhabha Cancer Hospital, Varanasi, IND; 2 Department of Microbiology, National Cancer Institute, Jhajjar, IND; 3 Department of Microbiology, All India Institute of Medical Sciences, Madurai, IND; 4 Department of Microbiology, All India Institute of Medical Sciences, New Delhi, IND

**Keywords:** antimicrobial susceptibility, pathogen identification, image analysis, microbial forensics, diagnostic microbiology, machine learning, artificial intelligence

## Abstract

Diagnostic microbiology plays a vital role in managing infectious diseases, combating antimicrobial resistance, and containment of outbreaks. During the fourth industrial revolution, when artificial intelligence (AI) became an essential part of our day-to-day lives, its integration into healthcare would further revolutionize our knowledge and potential. Although in the budding stage, AI with machine learning is being increasingly utilized in various aspects of diagnostic microbiology. It can handle large datasets that are difficult to analyze manually. Researchers have developed and demonstrated several machine-learning algorithms for interpreting bacterial cultures, conducting image analysis for microbial detection, and predicting antimicrobial susceptibility patterns. Thus, AI may most likely be the ultimate solution to the ever-increasing demand for improved results with shorter turnaround times. AI can also assist forensic microbiologists in crime scene investigations, as it can guide individual identification, cause and time since death, and manner of death. This review summarizes the application of AI in diagnostic microbiology for performing diverse sets of microbial investigations and is an essential aid in forensic microbiology.

## Introduction and background

The world has been blessed in the past two decades with incredible technological advancements that have improved safety and quality of life. One of these advancements is using artificial intelligence (AI) in various fields, including medical disciplines. AI is a branch of science (mainly computer science) in which machines are made to imitate human mental faculties, empowering them with thinking, analytical, and decision-making power [1]. Multiple applications developed on this concept are frequently used in our day-to-day routines, like Google Assistant, Siri, and Alexa, and have been great problem solvers in certain challenging situations.

The term medical AI can be used for the computational programs and technologies that help medical personnel diagnose quickly and promptly and choose easy and best therapeutic options [2]. AI has a crucial role in diagnostic or laboratory medicine, from developing a completely automated diagnostic system to the daily handling of the so-called *big data* [3]. In this era of technology, laboratories are dealing with the accumulation of vast amounts of complex data (called *big data*) whose proper analysis and interpretation may at times be beyond the mental capabilities of a human being and thus would require assistance from a machine or computer [4].

In microbiology laboratories, this *big data* may include the genomic data from sequencing or the large digital images produced from mass spectrometry [4]. AI tools for analyzing such massive data may help create a better quality laboratory report by minimizing the scope of human error and increasing the speed of analysis (reducing turnaround time) [5]. Although human intelligence and knowledge cannot be replaced by AI in diagnostic microbiology, combining these will lead to a faster, better, and more accurate output of services even with limited resources.

## Review

History of AI

The concept of AI dates back to the 1940s and 1950s when a young British polymath, Alan Turing, explored the idea that machines can be developed into smart devices and that their intelligence may be tested. He discussed the above two issues in his article *Computing Machinery and Intelligence*, in which he gave the concept of the *Turing Test*, published in 1950. This concept may still hold to date as a benchmark to identify the intelligence of a machine or an artificial system and states that if a human is interacting with another human and a machine and is unable to distinguish the machine from the human, then the machine is said to be intelligent [5,6].

The year 1956 marks the historical event of the Dartmouth Summer Research Project on Artificial Intelligence (DSRPAI) at Dartmouth College in New Hampshire, where John McCarthy and Marvin Minsky introduced the term *AI* [6,7]. Also, in this event, a program known as Logic Theorist, conceptualized by Allen Newell, Cliff Shaw, and Herbert Simon, was presented. This program, considered the first AI program by many researchers, simulated a human's problem-solving mental abilities [5].

**Figure 1:**
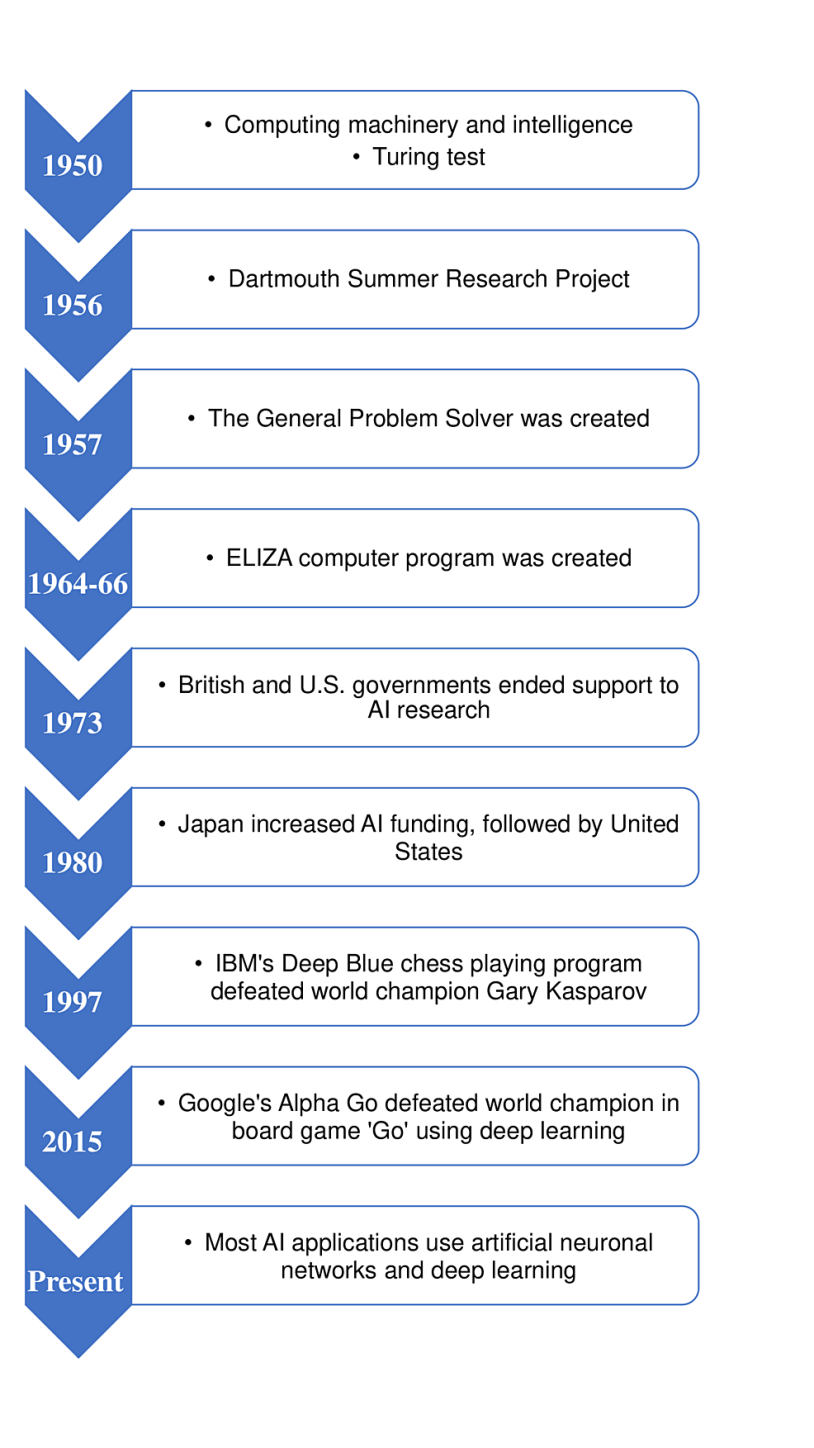
Brief timeline of developments of AI. Source: [6]. AI, artificial intelligence; US, United States

From the 1960s to the 1980s, AI concepts, research, and development went through multiple ups and downs to the revolutionary phase in the 1990s and 2000s, where the most significant goals and landmark developments were made in this field [5,6]. Figure 1 depicts the brief timeline of AI since its inception till date.

AI in healthcare and microbial diagnosis

There are three crucial techniques by which AI works, namely, machine learning, which, in turn, includes neural networks and support vector machines. The other two techniques include deep learning and natural language processing (NLP). Among these, the machine-learning technique is the predominant form of AI used in the medical discipline, primarily for structured data generated from hospital laboratories. The machine-learning process may be categorized into supervised, unsupervised, and reinforced learning. Deep learning and neural networks are complex machine-learning variants predominantly used in onco-radiology [2]. The third technique of AI, called NLP, mainly helps in the creation and maintenance of hospital electronic media records (EMRs), which later can provide valuable information for diagnosing various diseases [8]. The major applications of the NLP include speech analysis, which may help develop a conversational form of AI and translate patient interactions, and text analysis, which can help analyze patients' clinical notes, health records, etc. Both deep learning and NLP are mainly used for unstructured data [2].

In a diagnostic microbiology laboratory, AI can be helpful in diverse activities like image analysis and screening of slides for pathogenic organisms, culture interpretations, and antimicrobial susceptibility testing and interpretation with some aid in identifying the mechanism responsible for antimicrobial resistance (AMR). AI systems also benefit the laboratories dedicated to molecular testing like sequencing or proteomics, as they help in the analysis, interpretation, and storage of the large volumes of complex data generated in these laboratories.

Interpretation of microbial culture

The most challenging application of AI in microbiology is to develop and analyze various computationally generated complex algorithms for culture identification. There are three main algorithms [4].

Chromogenic Media Image Detection

The computational image analysis technique is used to study the surveillance cultures. Few studies have shown the advantage of this technique in detecting positive cultures with high sensitivity and specificity, particularly in laboratories receiving high volumes of these cultures with less workforce. The limitation of subjective variation due to human error may also be avoided by using this AI technique [3].

Colony Counting With Growth Versus No-Growth Discrimination

This algorithm best applies to samples that may be received in bulk in the laboratory. For example, the urine sample is one such sample as urinary tract infection (both community-acquired and hospital-acquired) is one of the most prevalent bacterial infections, especially in females. In fully automated laboratories, software algorithms can be applied to differentiate negative urine cultures from those with growth over and above the threshold set by the laboratory [3]. At present, the only class II medical device system with United States Food and Drug Administration (FDA) clearance is Automated Plate Assessment System (APAS) Independence (Clever Culture Systems). These systems can exclude the negative or no growth cultures in urine sample culture analysis while also playing an essential role in excluding insignificant growth when used for methicillin-resistant *Staphylococcus aureus* (MRSA) detection. A similar application system is the PhenoMatrix (bioMérieux, Craponne, France), with published studies on group B *Streptococcus* and *Streptococcus pyogenes* [9]. Recently, Becton Dickinson Kiestra developed a deep convolutional neural network (CNN) for culture analysis by capturing the images of urine samples [10].

Phenotypic Colony Recognition and Application of Expert Rules

Recent studies have shown that the analysis of culture plates using applications based upon expert image analysis systems can be used as a screening technique to help in an easy and quick reading of culture plates and better differentiate the positive from negative cultures. Cultures yielding no growth and cultures contaminated with normal flora would be excluded from further analysis and processing, leading to reduced manual work and increased efficiency of the available human resources [3]. Although accurate assessment by AI system for the contamination in samples is a highly challenging task and may not be entirely possible as this decision depends upon several factors like the type of specimen, time passed in transit and processing, history, and clinical presentation of the patient, and lastly on the judgment of the expert microbiologist. Therefore, expecting an AI system for such an extensive analysis may be difficult [9].

Identification of pathogens by image analysis

Another application of AI called *computer vision*, for example, CellaVision (Lund, Sweden) can be used to analyze various large and complex images and compare them with the existing databases, thus helping identify rare or difficult-to-identify microorganisms like acid-fast bacilli or malarial parasites. The software based on *computer vision* can act as an excellent screening tool by studying large and complicated images in less time and with less effort. As with all AI-based algorithms, this technique requires an expert human analysis of complex image patterns for final diagnosis and reporting. A recent study by Mathison et al. has shown computer vision as a valid AI technique for identifying and detecting protozoal parasites and helminthic ova in trichrome-stained fecal smears [11]. This study focuses on the easy exclusion of possible negative fecal smears for the concerned parasite, thus limiting the analysis of only the positive slides by an expert. As the extra labor of screening negative slides is reduced, there is an improved likelihood of parasite detection and characterization [4].

Prediction of susceptibility to antimicrobial agents

AI plays a significant role not only in the diagnosis of pathogenic microorganisms but also in assessing their antimicrobial susceptibility pattern. Recent studies have demonstrated the use of AI algorithms to detect resistance to aminoglycosides in bacteria like *Escherichia coli* and *S. aureus* [12]. Computer vision technology can assess the screening cultures for drug-resistant bugs like vancomycin-resistant *Enterococcus* (VRE) or MRSA. This may help their early detection and timely intervention for their treatment and control [4].

Total laboratory automation that uses principles of AI is being increasingly installed at multiple large-scale laboratories for microbial identification and antimicrobial susceptibility testing. Two primary total laboratory automation setups are the Kiestra TLA system (Becton Dickinson, Franklin Lakes, NJ, USA) and WASPLab (Copan Diagnostics Inc., Murrieta, CA, USA) [13].

Over the last decade, the matrix-assisted laser desorption ionization-time-of-flight mass spectrometry (MALDI-TOF MS) technology has been widely used to rapidly and accurately diagnose microorganisms. The diagnostic application of MALDI-TOF MS could be significantly improved by combining it with AI and machine learning not just for the identification of microorganisms but also for the prediction of AMR. Feucherolles et al. combined MALDI-TOF MS with machine learning analysis using Python programming language to effectively detect AMR in *Campylobacter* species with maximum sensitivity and precision of 92.3% and 81.2%, respectively [14].

Genomic sequence analysis and bioinformatics

High-throughput molecular methods and genomic sequencing technologies have revolutionized the field of microbiology as they allow detailed study of microorganisms like genetic relationships, detection of mutations, AMR genes, etc. But they have also led to the rapid expansion of biological data, which would only be possible to analyze with conventional methods. AI and machine learning algorithms are effectively utilized for this purpose. Sandberg et al. developed a naïve Bayesian classifier that could correctly classify sequences as short as 400 bases with 85% accuracy [15]. AI can also be used for data integration for the human gut microbiome and thereby help predict microbiome-related diseases, manipulating the gut microbiome as a potential therapeutic strategy [16]. Sharma et al. developed *Woods*, an orthology-based functional classifier combining machine learning and similarity-based approaches. It displayed a precision of 98.79% and performed >87 times faster than the Basic Local Alignment Search Tool (BLAST) on the two real metagenomic datasets [17]. Nguyen et al. developed and tested a machine-learning model to predict antimicrobial minimum inhibitory concentrations and associated genomic features of nontyphoidal Salmonella with up to 95% accuracy [18].

Microbial forensics

Forensic microbiology or microbial forensics is a subdiscipline that utilizes microbiological expertise to assist forensic investigations. Application of forensic microbiology may range from accidental release of biological agents, frauds, biocrimes, bioterrorism, etc. [19]. This field gained importance in 2001 after the Bacillus anthracis bioterror attack through the postal services of the United States was solved using high-resolution whole-genome sequencing and comparative genomics [20]. Forensic microbiology is primarily aimed at microbial source detection to investigate the postmortem interval (PMI), identification of the individuals, tissue/fluid identification, crime locations, and ultimately cause of death. AI with machine learning already started transforming the field of forensic microbiology. Microbial succession during postmortem decay can be a promising tool for PMI detection [21]. Johnson et al. have developed an AI algorithm to effectively investigate PMI by taking skin microbiota samples from nasal and ear canals [22]. Microbiome characterization can be a potential tool for performing one of the most critical tasks of individual identification, as, theoretically, every individual carries a unique set of microbial communities that remains relatively stable over long periods [21]. Yang et al. identified individuals with an accuracy of 90% using random forest machine learning by integrating Cutibacterium acnes 16S rRNA genotype with skin microbiome profile data [23]. Attempts have also been made to identify various tissues/body fluids based on microorganism composition, as these could greatly help during crime reconstruction. Using taxonomy-independent deep learning networks, Díez López et al. demonstrated high body-site classification accuracy in oral, skin, and vaginal secretions [24]. The Metagenomics and Metadesign of the Subways and Urban Biomes (MetaSUB), an interdisciplinary initiative, was launched in the year 2015 with the ultimate aim of enhancing the utilization and planning of cities by detecting, evaluating, and designing urban environmental metagenomics [25]. Huang et al. accessed data from MetaSUB and showed a good accuracy of 86% in demonstrating the geographical origin of metagenomic samples using machine learning by applying logistic regression [26]. Diatom examinations have classically been used in drowning investigations to discover the cause of death. Zhou et al. adopted an AI-based approach to automatically identify diatoms by training CNN models using transfer learning and data augmentation methods [27]. Forensic microbiology is still in the budding stage, with further utilization of large datasets provided by gene sequencing that will strengthen this field.

There are limitations to AI-generated reports in the judicial process. These include inexplicability, as there lies a *black box* of algorithmic calculations between the method of data entry and obtaining conclusions that may be inaccessible or difficult to understand in the courtroom; discrimination bias due to incomplete or faulty data from mistakes or cyberattacks; and lack of accountability since machine learning systems evolve in unforeseen ways due to their self-learning mechanism and hence programmers could be held liable for some wrong happening [28]. Therefore, there are enormous challenges ahead for judicial operators in evaluating the admissibility of such evidence due to concerns related to reliability, transparency, interpretability, and bias [29].

Challenges and the way forward

While AI can be highly beneficial, various challenges lie ahead that need to be addressed. AI may interpret single organism polymorphisms that are phenotypically distinct as separate organisms [9]. Likewise, it is challenging to expect an AI system to accurately recognize *contamination* because this judgment frequently requires years of experience, knowledge of the specimen type, and comprehension of the whole clinical picture with thorough clinical histories. Another challenge for AI in microbiology is interpretation of antimicrobial susceptibility testing data. Several classes of antimicrobials have diverse but frequently overlapping mechanisms of action. Resistance to multiple antimicrobials often coexist in the same organism.

On the other hand, many antibiotics have synergistic actions on organisms. Making fixed programs and standardized algorithms by considering all these aspects may be challenging in predicting an organism's susceptibility to antimicrobials. Furthermore, data are often imperfect irrespective of the source, which may introduce noise. Steps need to be taken for data cleaning to mitigate these noises.

Despite these challenges, the day is not far when AI will become indispensable in diagnostic Microbiology laboratories. More powerful AI products and algorithms with stable performances and convenient usage must be developed to benefit patients and medical staff in terms of reduced expenses, turnaround time, and minimum errors. Education and training on AI-based technologies for technicians need to be enhanced. Efforts must also be made to bring these technologies to the peripheral and remote areas.

## Conclusions

AI would revolutionize diagnostic microbiology by improving the accuracy and precision of microbial identification, effectively predicting microbial susceptibility to antimicrobials. Similarly, it can be applied in microbial forensics to successfully investigate bioterrorism attacks and crime scenes. As this technology is still in the nascent phase of development, the challenges of this evolving technology need to be addressed before their integration into Microbiology laboratories on a routine basis. The limitations, like the misinterpretation of single organism polymorphisms, the inability to differentiate true pathogens from contaminants, and the unavailability of fixed algorithms that could integrate multiple mechanisms of drugs and numerous resistance mechanisms possessed by microorganisms, need attention. Although AI appears attractive and promising for promptly diagnosing infectious diseases and predicting treatment responses to antimicrobials, it should only partially replace human intervention in a microbiology laboratory.
